# Association of remnant cholesterol with hypertension, type 2 diabetes, and their coexistence: the mediating role of inflammation-related indicators

**DOI:** 10.1186/s12944-023-01915-y

**Published:** 2023-09-26

**Authors:** Yuxuan Wu, Qinfei Wei, Husheng Li, Han Yang, Yuying Wu, Yiming Yu, Qiansi Chen, Baochang He, Fa Chen

**Affiliations:** 1 Department of Epidemiology and Health Statistics, School of Public Health, Fujian Medical University, Fuzhou, China; 2 Department of Preventive Medicine, School of Public Health, Fujian Medical University, Fuzhou, China; 3 Clinical Research Unit, The Second Affiliated Hospital, Fujian Medical University, Quanzhou, China

**Keywords:** Hypertension, Type 2 diabetes, Comorbidity, Remnant cholesterol, Inflammation

## Abstract

**Purpose:**

Cholesterol metabolism is a risk factor for cardiovascular disease, and recent studies have shown that cholesterol metabolism poses a residual risk of cardiovascular disease even when conventional lipid risk factors are in the optimal range. The association between remnant cholesterol (RC) and cardiovascular disease has been demonstrated; however, its association with hypertension, type 2 diabetes mellitus (T2DM), and the concomitance of the two diseases requires further study. This study aimed to evaluate the association of RC with hypertension, T2DM, and both in a large sample of the U.S. population, and to further explore the potential mechanisms involved.

**Methods:**

This cross-sectional study used data from the 2005—2018 cycles of the National Health and Nutrition Examination Survey (*N* = 17,749). Univariable and multivariable logistic regression analyses were performed to explore the relationships of RC with hypertension, T2DM, and both comorbidities. A restricted cubic spline regression model was used to reveal the dose effect. Mediation analyses were performed to explore the potential mediating roles of inflammation-related indicators in these associations.

**Results:**

Of the 17,749 participants included (mean [SD] age: 41.57 [0.23] years; women: 8983 (50.6%), men: 8766 (49.4%)), the prevalence of hypertension, T2DM, and their co-occurrence was 32.6%, 16.1%, and 11.0%, respectively. Higher RC concentrations were associated with an increased risk of hypertension, T2DM, and their co-occurrence (adjusted odds ratios for per unit increase in RC were 1.068, 2.259, and 2.362, and 95% confidence intervals were 1.063–1.073, 1.797–2.838, and 1.834–3.041, respectively), with a linear dose–response relationship. Even when conventional lipids were present at normal levels, positive associations were observed. Inflammation-related indicators (leukocytes, lymphocytes, monocytes, and neutrophils) partially mediated these associations. Among these, leukocytes had the greatest mediating effect (10.8%, 14.5%, and 14.0%, respectively).

**Conclusion:**

The results of this study provide evidence that RC is associated with the risk of hypertension, T2DM, and their co-occurrence, possibly mediated by an inflammatory response.

**Supplementary Information:**

The online version contains supplementary material available at 10.1186/s12944-023-01915-y.

## Introduction

It is universally acknowledged that hypertension and type 2 diabetes (T2DM) are two of the most common chronic non-communicable diseases worldwide, with an estimated 1.28 billion and 422 million people worldwide suffering from hypertension and T2DM, and they have a serious impact on the structure and function of vital organs [1, 2]. Elevated blood pressure and high glucose levels are major contributors to the increased burden of chronic diseases and mortality [3, 4]. As two interrelated metabolic disorders, the concomitance of hypertension and T2DM is twice as common as the incidence of the single disease [5], which could markedly increase the risk of micro- and macrovascular complications such as retinopathy, nephropathy, acute coronary syndrome, and stroke [6]. It is necessary to continue to identify modifiable risk factors for hypertension, T2DM, and the coexistence of the two diseases.

Accumulating studies have now found that physical inactivity, overweight, and dyslipidemia may all be possible risk factors for hypertension and T2DM [7]. Among these factors, cholesterol metabolism was in a pivotal position in the development of the two diseases [8]. Most previous studies regarding cholesterol metabolism have focused on low-density lipoprotein cholesterol (LDL-C) and high-density lipoprotein cholesterol (HDL-C), whereas the latest studies indicate that even when conventional risk factors such as LDL-C and HDL-C are in the optimal range, cholesterol metabolism still carries a residual risk of cardiovascular disease [9, 10]. Much attention, therefore, should be directed to the “bad cholesterol,” which is the residual cholesterol (RC), in addition to LDL-C. RC, also known as non-LDL-C and non-HDL-C, is a type of cholesterol that remains in the bloodstream after the body has finished using cholesterol for various physiological processes [11]. To date, a small number of preliminary researches have reported that RC is a possible risk factor for hypertension and T2DM [12, 13]. However, these relatively few studies mainly focus on a single disease rather than considering the concomitance of the two diseases. This encouraged us to explore the association between RC and the risk of hypertension, T2DM, and the co-occurrence of the two diseases in a large study.

The mechanisms underlying the effects of RC on hypertension and T2DM remain unclear. Moderate evidence has shown that RC may accumulate in the vessel wall, activating monocytes and leukocytes, ultimately leading to inflammation of the arterial wall and a multi-level cellular immune response [14]. Given that the inflammatory response is typically thought to be fundamental to the development of hypertension and diabetes [15, 16], this study hypothesized that inflammation-related responses may emerge as an important player in the relationship between RC and these two diseases.

Thus, the objective of this study was to evaluate the association between RC and hypertension, T2DM, and the co-occurrence of the two diseases using data from the National Health and Nutrition Examination Survey (NHANES) conducted between 2005 and 2018 and further explore the potential mediating role of inflammation-related indicators in that relationship with a goal provide a more informative basis for clinical practice.

## Methods

### Study population

This study downloaded data from the NHANES to perform a cross-sectional study. NHANES is a publicly available database of a stratified, multi-stage probability sample of American residents [17, 18]. Seven of these cycles (2005–2018) were selected as the data for the analysis.

**Fig. 1:**
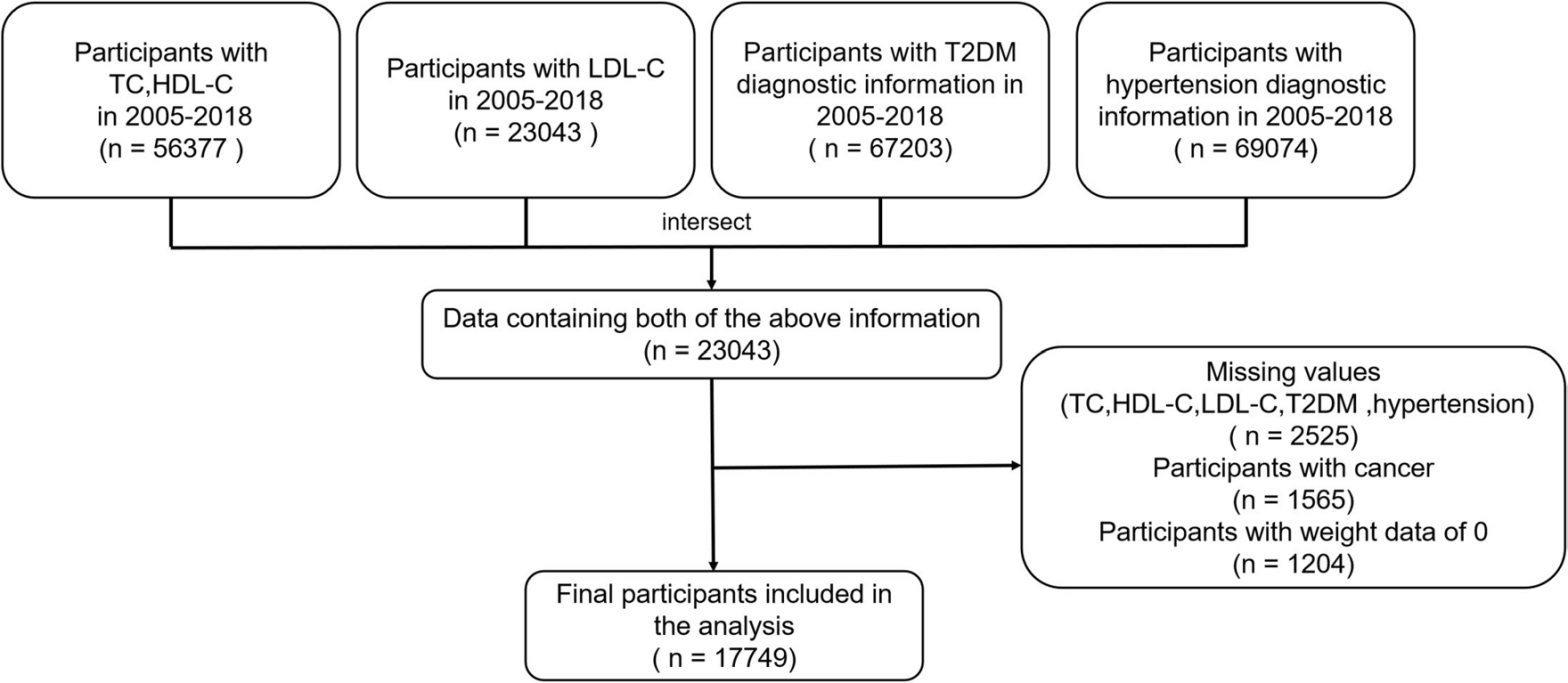
Flowchart of study (NHANES 2005–2018, *N* = 17,749)

Participants with complete total cholesterol (TC), LDL-C, and HDL-C data from the NHANES 2005–2018, as well as those with comprehensive diagnostic information on hypertension and T2DM, were selected. Patients with cancer or those with a weight value of zero were excluded. In total, 17,749 participants were integrated into the final analysis (Fig. 1). All patients signed an informed consent form, and following the principles of the Declaration of Helsinki, the study did not require ethical review by Fujian Medical University.

### Definition of lipid-related indicators

Serum or plasma TC, HDL-C, and LDL-C were measured in participants who fasted for more than 8.5 h and less than 24 h in the NHANES laboratory. Specifically, TC was measured using enzymatic assays and HDL-C was measured using immunoassays. LDL-C was calculated from the measured values of total cholesterol (TC), triglycerides, and HDL-C according to the Friedewald calculation: [LDL-C] = [TC]—[HDL-C]—[triglycerides/5]. The timed-endpoint method was used to determine the concentration of triglycerides in serum or plasma. The 2019 European Society of Cardiology and European Atherosclerosis Society (ESC/EAS) guidelines for the management of dyslipidaemias recommend the use of TC minus HDL-C and LDL-C for RC calculation [19]. The normal level of blood lipids were defined as TC < 5.18 mmol/L, LDL-C < 3.37 mmol/L, and HDL-C ≥ 1.04 mmol/L [20].

### Outcome ascertainment

Hypertension [21, 22]: In previous studies based on NHANES [23, 24], hypertension was usually diagnosed with three times measurements of blood pressure, including systolic blood pressure (SBP) and diastolic blood pressure (DBP), using a mercury sphygmomanometer at a mobile examination center (MEC). The methodology followed the latest recommendations of the American Heart Association for determining blood pressure in humans by sphygmomanometer, and the average of the three blood pressure measurements was used for analysis. SBP ≥ 140 mm Hg or DBP ≥ 90 mm Hg was considered hypertension, while SBP < 140 mm Hg and DBP < 90 mm Hg were considered non-hypertension. In this study, we also used the above methods as one of the diagnostic methods for hypertension. In addition, we also used the following two criteria: (1) told had high blood pressure by a doctor; (2) taking prescriptions for hypertension. If one of these three criteria mentioned above was met, the participants were considered to have hypertension. T2DM [25, 26]: We diagnosed T2DM according to (1) told had T2DM by a doctor. (2) hemoglobin A1C (Hba1c) ≥ 6.5%. (3) fasting blood glucose ≥ 7.0 mmol/L. (4) random glucose ≥ 11.1 mmol/L. (5) Oral Glucose Tolerance Test (OGTT) ≥ 11.1 mmol/L. (6) Whether using diabetes medication. T2DM was diagnosed when one of the above six conditions was met.

### Covariates

Age, sex, race, marital status, education, and poverty income ratio (PIR) were selected as demographic variables. The relevant covariates for behavioral habits and physical indicators included smoking, current alcohol consumption, body mass index (BMI), waist circumference, healthy eating index (HEI), and dietary inflammation index (DII). Specific definitions of covariates are detailed in the supplementary material.

Smoking status was defined as follows: never (less than 100 cigarettes smoked in their lifetime), former (had smoked in the past but had quit at the time of the interview), current (had smoked at least 100 cigarettes throughout their lifetime and continued to smoke cigarettes currently) [27]. We define current drinking status as “yes” or “no” (no was defined as having fewer than 12 drinks ever or having ≥ 12 drinks in 1 year but did not drink in the previous year or did not drink last year but drank 12 drinks in lifetime) [28]. BMI was calculated by dividing weight in kilograms by height in meters squared and divided into normal (< 25 kg/m^2^), obese (25–30 kg/m^2^), and overweight (≥ 30 kg/m^2^) according to the World Health Organization [29]. PIR is an indicator of poverty status, calculated by dividing the total household income by the poverty threshold [30]. HEI is a measure for assessing whether a set of foods aligns with the Dietary Guidelines for Americans (DGA) and includes 13 food components [31]. The latest 2015 version of the DGA was used in this study. DII is a literature-derived dietary tool used to assess the overall inflammatory potential of an individual's diet [32].

Blood indicators such as white blood cell count, neutrophil count, and monocyte count in NHANES 2005–2018 were detected using the Beckman Coulter MAXM instrument in the Mobile Examination Centers. Segmented neutrophils count (1000 cells/μL) = white blood cell count × segmented neutrophils percent (%)/100. Lymphocyte count (1000 cells/μL) = white blood cell count × Lymphocyte percent (%)/100. Monocyte count (1000 cells/μL) = white blood cell count × monocyte percent (%)/100. In addition, we use a number of ratio indicators, such as NLR, neutrophil to lymphocyte ratio (neutrophil/lymphocyte); PLR, platelet to lymphocyte ratio (platelet/lymphocyte); LMR, lymphocyte to monocyte ratio (lymphocyte/monocyte).

### Statistical analyses

As NHANES uses a complex multi-stage stratified sampling design, we weighted the data by the "Fasting Subsample 2 Year MEC Weight,” and as the data we used had seven cycles, we divided the weight value by 7 to construct the sampling weights for the 7-year cycle combination [33].

To explore the relationship between RC and hypertension, T2DM, and both comorbidities, we performed univariable and multivariable logistic regression, with adjustment for covariates in the multivariable logistic regression model, including age, sex, race, education, marital status, PIR, current alcohol drinking, smoking, BMI level, HEI, DII, and waist circumference. The shapes of the relationships between RC and hypertension, T2DM, and their coexistence were explored using a restricted cubic spline regression model. In addition, we performed a subgroup analysis to analyze the association between RC and hypertension, T2DM, and both comorbidities when blood lipid levels were normal.

Interaction analyses were performed first, and stratified analyses were then performed only if there was an interaction. Considering that Age, HEI, DII, PIR, and waist circumference are continuous variables, they were not subjected to stratified analysis. Mediation analyses were used to explore the mediating effects of inflammation-related indicators (leukocytes, neutrophils, lymphocytes, and monocytes) on the relationships between RC and hypertension, T2DM, and their coexistence (adjustment for all confounding variables). The Appendix Fig. 1 showed the schematic of a simple mediation model. The following criteria were defined as having a mediating effect: (1) the total effect was significant, (2) the indirect effect was significant, and (3) the proportion of mediators was positive [34]. We performed 5000 bootstrap mediations using the "mediation" package.

In the sensitivity analysis section, we performed the same correlation analysis for data with multiple interpolations (based on the Mice package) and data in other units (mg/dL). In the analysis of the coexistence module, we divided the population into four groups (no hypertension and no T2DM, only hypertension, only T2DM, hypertension & T2DM) and performed unordered multivariable logistic regression. A two-tailed *P* < 0.05 was considered statistically significant. R (version 4.2.1) was used for all the analyses.

## Results

### Characteristics of participants

The 17,749 participants in this study included 5,791 with hypertension only, 2,857 with T2DM only, and 1,958 with both (Table 1). The average age of the participants was 41.57 years, and the number of men was 8983 (50.6%) and women was 8766 (49.4%). Age, HEI, PIR, DII, waist circumference, race, education, marital status, smoking history, current alcohol consumption, and BMI were significantly different between the control and hypertension, T2DM, and coexistence groups (*P* < 0.05).

**Table 1:** Demographic characteristics of the total population in NHANES 2005–2018 (*N* = 17,749) *

Variables	Total *N* = 17,749	Non-T2DM *N* = 14,892 (83.9%)	T2DM *N* = 2857(16.1%)	*P* value	Non-hypertension *N* = 11,958(67.4%)	Hypertension *N* = 5791(32.6%)	*P* value	Non-hypertension& T2DM *N* = 15,791(89.0%)	Hypertension& T2DM *N* = 1958(11.0%)	*P* value
Age (years)	41.57(0.23)	39.24(0.24)	57.63(0.37)	< 0.001	35.56(0.23)	55.10(0.30)	< 0.001	39.79(0.23)	60.63(0.36)	< 0.001
Healthy diet index (HEI)	49.22(0.22)	49.01(0.25)	50.70(0.40)	< 0.001	48.88(0.26)	49.97(0.31)	0.003	49.04(0.24)	51.18(0.45)	< 0.001
Poverty-income ratio (PIR)	2.89(0.04)	2.92(0.04)	2.70(0.05)	< 0.001	2.89(0.04)	2.88(0.04)	0.703	2.91(0.04)	2.68(0.07)	0.001
Dietary inflammation index (DII)	1.55(0.03)	1.52(0.04)	1.69(0.05)	0.004	1.54(0.04)	1.55(0.04)	0.881	1.53(0.03)	1.71(0.06)	0.004
Waist	96.47(0.25)	94.54(0.23)	110.05(0.55)	< 0.001	92.45(0.26)	105.69(0.34)	< 0.001	95.06(0.23)	112.06(0.61)	< 0.001
Sex				0.632			0.222			0.579
Female	8983(50.6)	7589(50.7)	1394(50.1)		6065(51.0)	2918(49.8)		8002(50.5)	981(51.4)	
Male	8766(49.4)	7303(49.3)	1463(49.9)		5893(49.0)	2873(50.2)		7789(49.5)	977(48.6)	
Race				< 0.001			< 0.001			< 0.001
Mexican American	3281(18.5)	2733(9.6)	548(10.5)		2515(11.2)	766(6.5)		2965(9.8)	316(8.3)	
Non-Hispanic Black	3904(22.0)	3207(11.8)	697(15.2)		2339(10.9)	1565(15.2)		3353(11.8)	551(17.2)	
Non-Hispanic White	6633(37.4)	5683(64.5)	950(59.2)		4318(62.9)	2315(65.9)		5966(64.2)	667(60.4)	
Other	3931(22.1)	3269(14.1)	662(15.1)		2786(15.0)	1145(12.4)		3507(14.2)	424(14.1)	
Education				< 0.001			< 0.001			< 0.001
High school	3547(20.0)	2865(20.6)	682(27.0)		2104(19.2)	1443(26.4)		3067(20.8)	480(27.8)	
Less than high school	6633(37.4)	5618(26.4)	1015(24.7)		4945(28.9)	1688(20.0)		5950(26.4)	683(23.9)	
Some college or above	7553(42.6)	6398(53.0)	1155(48.3)		4901(51.9)	2652(53.6)		6762(52.8)	791(48.3)	
Marital status				< 0.001			< 0.001			< 0.001
Married	7270(49.6)	5693(52.7)	1577(58.5)		4270(51.6)	3000(57.2)		6195(53.1)	1075(57.7)	
Never married	3217(21.9)	2947(22.5)	270(9.8)		2528(25.5)	689(11.6)		3048(21.9)	169(9.0)	
Unmarried but have/had partner	4178(28.5)	3209(24.8)	969(31.7)		2157(22.9)	2021(31.2)		3470(25.0)	708(33.3)	
Smoking				< 0.001			< 0.001			< 0.001
Former	3264(22.7)	2387(22.2)	877(32.3)		1611(20.2)	1653(30.0)		2613(22.3)	651(35.3)	
Never	8249(57.4)	6779(57.2)	1470(50.9)		5296(59.6)	2953(50.2)		7251(57.1)	998(49.3)	
Now	2861(19.9)	2391(20.6)	470(16.8)		1770(20.2)	1091(19.8)		2558(20.6)	303(15.4)	
Current alcohol drinking				< 0.001			< 0.001			< 0.001
No	3937(30.2)	2871(21.8)	1066(36.9)		2044(21.0)	1893(29.4)		3167(22.3)	770(39.0)	
Yes	9107(69.8)	7659(78.2)	1448(63.1)		5864(79.0)	3243(70.6)		8133(77.7)	974(61.0)	
BMI levels				< 0.001			< 0.001			< 0.001
Normal	6439(36.7)	6010(38.9)	429(12.9)		5354(43.5)	1085(18.1)		6186(38.0)	253(10.9)	
Obese	5849(33.4)	4306(30.1)	1543(60.0)		3023(26.1)	2826(51.2)		4721(31.0)	1128(64.1)	
Overweight	5248(29.9)	4426(31.0)	822(27.1)		3470(30.4)	1778(30.7)		4719(31.0)	529(25.0)	

*Abbreviations: T2DM* Type 2 diabetes, *BMI* Body mass index

^*^Continuous variables are represented as mean (SD), and classification variable is represented as n (%)

### Association of RC with hypertension, T2DM, and their coexistence

In the unadjusted model, RC was significantly associated with an increased risk of hypertension, T2DM, and the coexistence of the two diseases (*P* < 0.05; Table 2). After adjusting for sociodemographic characteristics, similar association patterns were observed. When further adjusted for smoking, alcohol consumption, BMI, HEI, DII, and waist circumference in model 4, the adjusted odds ratios (ORs) and 95% confidence intervals (CIs) for per unit increase in RC were 1.068 (1.063, 1.073) for hypertension, 2.259 (1.797, 2.838) for T2DM, and 2.362 (1.834, 3.041) for both comorbidities.

**Table 2:** Association between RC and hypertension, T2DM, and their coexistence in NHANES 2005–2018 (*N* = 17,749)

	**Unadjusted**		**Adjusted Model 1**		**Adjusted Model 2**		**Adjusted Model 3**	
	**OR (95% CI)**	***P* value**	**OR (95% CI)**	***P* value**	**OR (95% CI)**	***P* value**	**OR (95% CI)**	***P* value**
Raw data with missing values								
Hypertension	3.829(3.321,4.415)	< 0.001	2.601(2.252,3.004)	< 0.001	2.697(2.317,3.139)	< 0.001	1.068(1.063,1.073)	< 0.001
T2DM	4.158(3.603,4.798)	< 0.001	3.342(2.817,3.964)	< 0.001	3.557(2.950,4.288)	< 0.001	2.259(1.797,2.838)	< 0.001
Hypertension &T2DM	4.107(3.478,4.849)	< 0.001	3.449(2.821,4.217)	< 0.001	3.787(3.045,4.710)	< 0.001	2.362(1.834,3.041)	< 0.001
Multiple Imputation based on mice								
Hypertension	3.829(3.321,4.415)	< 0.001	2.601(2.252,3.004)	< 0.001	2.914(2.526,3.362)	< 0.001	1.866(1.594,2.186)	< 0.001
T2DM	4.158(3.603,4.798)	< 0.001	3.342(2.817,3.964)	< 0.001	3.594(3.002,4.304)	< 0.001	2.466(2.030,2.996)	< 0.001
Hypertension & T2DM	4.107(3.478,4.849)	< 0.001	3.449(2.821,4.217)	< 0.001	3.900(3.171,4.797)	< 0.001	2.661(2.148,3.297)	< 0.001
Unit replaced with mg/dL								
Hypertension	1.035(1.032,1.039)	< 0.001	1.025(1.021,1.029)	< 0.001	1.026(1.022,1.030)	< 0.001	1.014(1.010,1.019)	< 0.001
T2DM	1.038(1.034,1.041)	< 0.001	1.032(1.027,1.036)	< 0.001	1.033(1.028,1.038)	< 0.001	1.021(1.015,1.027)	< 0.001
Hypertension & T2DM	1.037(1.033,1.042)	< 0.001	1.033(1.027,1.038)	< 0.001	1.035(1.029,1.041)	< 0.001	1.022(1.016,1.029)	< 0.001

*Notes:* Model 1 was adjusted by age and sex; Model 2 was adjusted by age, sex, race, education, marital status, and PIR; Model 3 was additionally adjusted by smoking, alcohol, BMI levels, HEI, DII, and waist

*Abbreviations: T2DM* Type 2 diabetes, *RC* Remnant cholesterol, *OR* Odds ratio, *CI* Confidence interval

**Fig. 2:**
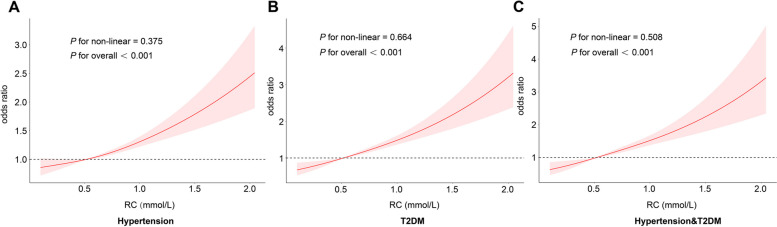
Restricted cubic spline curves of remnant cholesterol concentrations in NHANES 2005–2018 (*N* = 17,749)**.** Notes: (**A**) Restricted cubic spline curves of remnant cholesterol in hypertension; (**B**) Restricted cubic spline curves of remnant cholesterol in T2DM; (**C**) Restricted cubic spline curves of remnant cholesterol in hypertension & T2DM. Adjusted by age, sex, race, education, marital status, PIR, smoking, alcohol, BMI levels, HEI, DII, and waist. Abbreviations: T2DM, type 2 diabetes; RC, remnant cholesterol

Additionally, as shown in Fig. 2, RC was positively associated with the risk of hypertension, T2DM, and the coexistence of both (*P *_*overall*_ < 0.05). The risk of disease increased gradually as the RC increased, although the *P*-values for nonlinearity did not reach statistical significance.

### Association of RC with hypertension, T2DM, and their coexistence with normal TC, LDL-C, HDL-C levels

As presented in Table 3, when TC was less than 5.18 mmol/L, RC showed significant positive associations with hypertension, T2DM, and both coexistences, the adjusted ORs and 95%CI were 2.085 (1.565, 2.777), 2.481 (1.920, 3.206), 3.186 (2.319, 4.376), respectively. When LDL-C and HDL-C were at normal levels, consistent positive associations persisted, the adjusted ORs and 95%CI were 1.984 (1.572, 2.504) and 1.797 (1.440, 2.241) for hypertension, 2.169 (1.703, 2.763) and 2.431 (1.828, 3.232) for T2DM, 2.590 (1.957, 3.428) and 2.412 (1.813, 3.209) for both coexistences.

**Table 3:** Association between RC and hypertension, T2DM, and their coexistence when TC, LDL-C and HDL-C are at normal levels in NHANES 2005–2018 (*N* = 17,749)

	**TC < 5.18mmol/L**		**LDL-C < 3.37mmol/L**		**HDL-C ≥ 1.04mmol/L**	
	**OR (95%CI)**	*P ***value**	**OR (95%CI)**	*P ***value**	**OR (95%CI)**	*P ***value**
Hypertension	2.085(1.565,2.777)	<0.001	1.984(1.572,2.504)	<0.001	1.797(1.440,2.241)	<0.001
T2DM	2.481(1.920,3.206)	<0.001	2.169(1.703,2.763)	<0.001	2.431(1.828,3.232)	<0.001
Hypertension & T2DM	3.186(2.319,4.376)	<0.001	2.590(1.957,3.428)	<0.001	2.412(1.813,3.209)	<0.001

*Notes: *Adjusted by age, sex, race, education, marital status, PIR, smoking, alcohol, BMI levels, HEI, DII, and waist

*Abbreviations: T2DM* Type 2 diabetes, *RC* Remnant cholesterol, *TC* Total cholesterol, *LDL-C* Low-density lipoprotein cholesterol, *HDL-C* High-density lipoprotein cholesterol, *OR* Odds ratio, *CI* Confidence interval

### Subgroup analysis

As shown in Appendix Table 3, the results of the interaction analysis showed that sex interacted with RC when T2DM or co-morbidities was the outcome and that RC was positively associated with the outcomes in both men and women (*P* < 0.05). When hypertension was the outcome, there was an interaction between race and RC (*P* < 0.05). A positive association was observed between RC and hypertension among the Non-Hispanic White and other groups. In addition, there was an interaction between BMI and RC for hypertension or co-morbidities as an outcome (*P* < 0.05).

### Mediating role of inflammation-related indicators

**Fig. 3:**
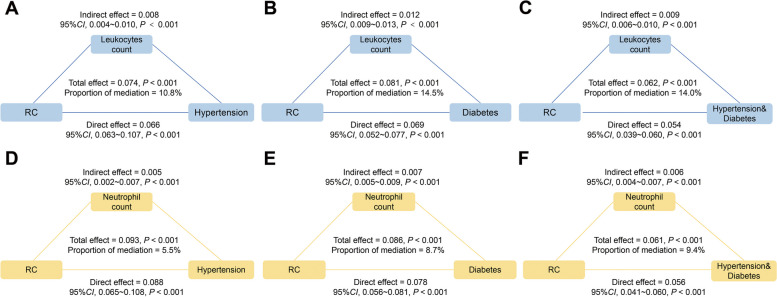
Analyses of the mediation by leukocytes and neutrophils of the association between RC and hypertension (**A**, **D**), T2DM (**B**, **E**), and the coexistence of the two diseases (**C**, **F**). Notes: Adjusted by age, sex, race, education, marital status, PIR, smoking, alcohol, BMI levels, HEI, DII, and waist. Abbreviations: T2DM, type 2 diabetes; RC, remnant cholesterol

Figure 3A-C shows that leukocytes mediated 10.8%, 14.5%, and 14.0% of the relationship between RC and hypertension, T2DM, and both comorbidities, respectively (*P* < 0.05). Similar results were observed when neutrophils were the mediating variables (Fig. 3D-F). Interestingly, a significant mediation effect of lymphocytes was found only for the association between RC and T2DM or both T2DM and hypertension, but not hypertension alone. Conversely, a significant mediating effect of monocytes was observed only for hypertension or both T2DM and hypertension, although no such effect was found for T2DM only (Appendix Table 4). In addition, we also analyzed the mediating effect of some ratio-based indicators (Appendix Table 4), PLR may partially mediate the association between RC and T2DM or co-morbidities (proportions of mediation were 0.98% and 1.46%, respectively, both *P* < 0.05).

**Table 4:** Multinomial logistic regression analysis of association between RC and hypertension, T2DM and their co-existence in NHANES 2005–2018 (*N* = 17,749)

**Variables**	**Unadjusted**		**Model 1**		**Model 2**		**Model 3**	
	**OR (95% CI) per unit**	*P*** value**	**OR (95% CI) per unit**	*P*** value**	**OR (95% CI) per unit**	*P*** value**	**OR (95% CI) per unit**	*P* value
No hypertension and no T2DM (*n*=11059)	ref	ref	ref	ref	ref	ref	ref	ref
Only hypertension (*n*=3833)	3.45 (3.08,3.87)	<0.001	2.03 (1.79,2.31)	<0.001	2.28 (1.98,2.63)	<0.001	1.55 (1.32,1.81)	<0.001
Only T2DM (*n*=899)	6.03 (5.05,7.21)	<0.001	3.71 (3.08,4.49)	<0.001	3.69 (2.98,4.56)	<0.001	2.45 (1.92,3.11)	<0.001
Hypertension & T2DM (*n*=1958)	6.58 (5.75,7.51)	<0.001	4.54 (3.88,5.32)	<0.001	5.65 (4.74,6.74)	<0.001	3.21 (2.61,3.94)	<0.001

*Notes:* Participants were classified into no hypertension and no T2DM, only hypertension, only T2DM, hypertension & T2DM

Model 1 was adjusted by age and sex; Model 2 was adjusted by age, sex, race, education, marital status, and PIR; Model 3 was additionally adjusted by smoking, alcohol, BMI levels, HEI, DII, and waist

*Abbreviations: T2DM* Type 2 diabetes, *RC* Remnant cholesterol, *OR* Odds ratio, *CI* Confidence interval

### Sensitivity analysis

Interpolation of the data using multiple imputations and subsequent analysis of the association between RC and hypertension, T2DM, or both yielded consistent results. Similar estimates were obtained when replacement units (mg/dL) were used (Table 2). Additionally, we further divided the population into four groups (no hypertension and no T2DM, only hypertension, only T2DM, hypertension & T2DM). As shown in Table 4, RC was positively associated with the risk of hypertension, T2DM, or both hypertension and T2DM (*P* < 0.05).

## Discussion

Our results showed that RC was associated with hypertension, T2DM, and both of these comorbidities. These positive associations remained even when TC, LDL-C, and HDL-C levels were normal and were more pronounced among women. Inflammation-related indicators partially mediated these associations.

Our study is consistent with most previous studies showing that high RC is associated with an increased risk of hypertension and T2DM [12, 35]. A cohort study found that RC in the blood, independent of other lipids, was significantly associated with an increase in systolic blood pressure [36]. The mechanisms by which RC contributes to hypertension remain unclear, although several mechanisms may be involved. Endothelial dysfunction can lead to vasoconstriction and thus increase blood pressure or lead to hypertension [37], whereas RC may lead to endothelial dysfunction by inducing intracellular oxidative stress in endothelial cells, leading to the inactivation of nitric oxide synthase [38]. In addition, residual lipoproteins can activate the epidermal growth factor receptor, leading to the proliferation of vascular smooth muscle cells [39, 40], and failure to maintain a normal differentiated phenotype of vascular smooth muscle cells results in poor vascular remodeling, which can also lead to hypertension [41].

A nationwide population-based cohort study found RC to be an independent predictor of T2DM [42]. RC, when degraded and metabolized by lipoprotein lipase, can produce free fatty acids and monoacylglycerols, causing an inflammatory response which in turn leads to diabetes [43]. Excess residual lipoprotein in plasma can penetrate the arterial wall and be taken up by macrophages and smooth muscle cells, leading to foam cell formation and low-grade inflammation that raises blood sugar [44, 45]. Moreover, RC is closely associated with insulin resistance [46], where arterial retention of residual lipoprotein isolates increases insulin resistance, leading to hyperglycemia [47, 48].

We found that a high RC may increase the risk of both comorbidities. A previous study found that RC significantly predicts hypertension in diabetic populations and that RC and T2DM interact in the subsequent risk of developing hypertension [49]. Similarly, a cohort study with a 9-year follow-up found stronger effects of RC on the comorbidity of hypertension and prediabetes than on each disease alone [50]. RC includes very-low-density lipoprotein cholesterol (VLDL-C), which can stimulate the release of aldosterone and increase circulating blood volume [51]. Available evidence suggests that both hyperactivity of the renin–angiotensin–aldosterone system and insulin resistance are potential mechanisms for hypertension complicated by diabetes [52, 53].

To date, the most commonly used clinical indicators of cholesterol to assess lipid levels are TC, LDL-C, and HDL-C; however, we found that even when these three indicators are at normal levels, RC still poses a risk for hypertension, T2DM, and their coexistence. Some studies have suggested that non-HDL-C has better utility than traditional cholesterol indices such as LDL-C in predicting diabetes risk [54]. Furthermore, elevated RC may lead to inflammation and coronary artery disease, whereas elevated LDL-C may only lead to coronary heart disease without an inflammatory response that performs a crucial role in the development of hypertension and T2DM [55]. Therefore, when conventional lipid levels are normal, we still need to be concerned about residual lipoprotein cholesterol in order to prevent its contribution to disease.

The results of the stratified interaction analysis showed a significant interaction between RC and sex for hypertension, T2DM, and their coexistence. Specifically, we observed more significant deleterious effects in women. This may be due to the stronger interaction of estrogen with cholesterol metabolism [56]. A current study found that RC increased the risk of cardiovascular disease in women [57]. Furthermore, the risk of metabolic syndrome associated with RC was significantly higher among women than among men [58]. These results may provide some support for our findings.

While exploring the relationship between RC and hypertension, T2DM, and their coexistence, we found that inflammation-related indicators (leukocytes, lymphocytes, monocytes, and neutrophils) played a partially mediating role. RC may contribute to the development of hypertension, T2DM, or both by stimulating an inflammatory response. Evidence has found that RC may promote monocyte activation, which in turn leads to endothelial dysfunction [59, 60]. Small dense LDL-C and remnant-like particle cholesterol have a combined effect on inflammation and may be associated with low-grade inflammation characterized by elevated C-reactive protein levels [61]. It has been found that VLDL-C and its component apolipoprotein C-III promote inflammatory responses by stimulating interleukin-1β [62]. In the progression of hypertension and T2DM, increased inflammatory cytokines impair insulin secretion and sensitivity and induce insulin resistance and β-cell dysfunction [63].

### Strengths and limitations

The strength of this study lies in its use of data from a large sample, full consideration of the risk factors involved, and adjustment for them. In addition, this study is the first to investigate the role of inflammation-related indicators in mediating the association of RC with hypertension, T2DM, and their coexistence in a nationally representative sample, providing initial clues regarding the mechanisms involved. However, this study has several limitations. Firstly, given this is a cross-sectional study, the causal association between RC and hypertension, T2DM, and both together could not be determined. Further cohort studies are needed to determine their association. Secondly, the NHANES database is a survey based on the U.S. population and has limitations in extrapolating results, and the collection of diseases and some covariates is obtained through questionnaires, thus potentially generating recall bias. Finally, RC levels were obtained using a formula and therefore have the potential to overestimate or underestimate the value of RC compared to direct measures. Additionally, the data on recent infections and antibiotic use was not available in the NHANES database, which precluded us from including them for adjustment in mediation analyses.

## Conclusion

This study suggests a significant positive relationship between RC values and the risk of hypertension, T2DM, and their coexistence. Notably, these positive associations persisted even when TC, LDL-C, and HDL-C levels were normal. Several inflammation-related indicators may partially mediate this association. Our findings indicate that RC can be used as a lipid predictor for hypertension, T2DM, and their co-occurrence, providing new insights into their prevention. This study has some clinical relevance: RC may serve as a clinical predictor of hypertension, T2DM, and their coexistence.

### Supplementary Information

**Additional file 1:** **Appendix Table 1.** The difference in the concentrations of RC, TC, HDL-C, LDL-C between different groups in NHANES 2005-2018 (*N*=17,749). **Appendix Table 2.** Demographic characteristics of the total population in NHANES 2005-2018 (*N*=17,749). **Appendix Table 3.** Interaction between baseline characteristics and RC in NHANES 2005-2018 (*N*=17,749). **Appendix Table 4. **Summary of simple mediation analyses for the relationships between mediator and outcomes, in NHANES 2005-2018 (*n* = 17749). **Appendix Figure 1. **Schematic of a simple mediation model.

## Data Availability

The data for this study is sourced from the National Health and Nutrition Examination Survey (NHANES), data can be downloaded for free from its official website (https://www.cdc.gov/nchs/nhanes/index.htm).

## Abbreviations

RC: Remnant cholesterol
T2DM: Type 2 diabetes mellitus
LDL-C: Low-density lipoprotein cholesterol
HDL-C: High-density lipoprotein cholesterol
NHANES: National Health and Nutrition Examination Survey
TC: Total cholesterol
PIR: Poverty income ratio
BMI: Body mass index
HEI: Healthy eating index
DII: Dietary inflammation index
ORs: Odds ratios
95%CIs: 95% Confidence intervals
VLDL-C: Very-low-density lipoprotein cholesterol
