# Supplementary material for: Association of remnant cholesterol with hypertension, type 2 diabetes, and their coexistence: the mediating role of inflammation-related indicators
Source: Lipids Health Dis. 2023 Sep 26;22:158. doi: 10.1186/s12944-023-01915-y (PMC10521406; doi:10.1186/s12944-023-01915-y)
Supplement: Supplementary file 1 — Additional file 1: Appendix Table 1. The difference in the concentrations of RC, TC, HDL-C, LDL-C between different groups in NHANES 2005-2018 (N=17,749). Appendix Table 2. Demographic characteristics of the total population in NHANES 2005-2018 (N=17,749). Appendix Table 3. Interaction between baseline characteristics and RC in NHANES 2005-2018 (N=17,749). Appendix Table 4. Summary of simple mediation analyses for the relationships between mediator and outcomes, in NHANES 2005-2018 (n = 17749). Appendix Figure 1. Schematic of a simple mediation model. [file 12944_2023_1915_MOESM1_ESM.docx]

**Appendix Table 1 The difference in the concentrations of RC, TC, HDL-C, LDL-C between different groups in NHANES 2005-2018 (N=17,749).**

| **Variable**  **Mean (SD)** | **Total**  **N = 17749** | **Non-T2DM**  **N = 14892 (83.9%)** | **T2DM**  **N = 2857(16.1%)** | ***P* value** | **Non-hypertension**  **N=11958(67.4%)** | **Hypertension**  **N=5791(32.6%)** | ***P* value** | **Non-hypertension&**  **T2DM**  **N=15791(89.0%)** | **Hypertension&**  **T2DM**  **N=1958(11.0%)** | ***P* value** |
| --- | --- | --- | --- | --- | --- | --- | --- | --- | --- | --- |
| **RC (mmol/L)** | 0.57 (0.00) | 0.55 (0.00) | 0.74 (0.01) | 0.081 | 0.52 (0.00) | 0.68 (0.01) | <0.001 | 0.55 (0.00) | 0.75 (0.01) | <0.001 |
| **Total cholesterol (mmol/L)** | 4.82 (0.01) | 4.83 (0.01) | 4.77 (0.03) | <0.001 | 4.74 (0.01) | 5.00 (0.02) | <0.001 | 4.83 (0.01) | 4.70 (0.04) | 0.001 |
| **LDL-C (mmol/L)** | 2.85 (0.01) | 2.87 (0.01) | 2.74 (0.03) | <0.001 | 2.81 (0.01) | 2.94 (0.02) | <0.001 | 2.87 (0.01) | 2.67 (0.03) | <0.001 |
| **HDL-C (mmol/L)** | 1.40 (0.01) | 1.42 (0.01) | 1.29 (0.01) | <0.001 | 1.41 (0.01) | 1.38 (0.01) | 0.001 | 1.41 (0.01) | 1.28 (0.01) | <0.001 |

**Abbreviations:** T2DM, type 2 diabetes.

**Appendix Table 2** Demographic characteristics of the total population in NHANES 2005-2018 (N=17,749)

| Variable  Mean (SD) | Total  N = 17749 | Non-T2DM  N = 14892 (83.9%) | T2DM  N = 2857(16.1%) | *P* value | Non-hypertension  N=11958(67.4%) | Hypertension N=5791(32.6%) | | *P* value | Non-hypertension&  T2DM  N=15791(89.0%) | Hypertension&  T2DM  N=1958(11.0%) | *P* value |
| --- | --- | --- | --- | --- | --- | --- | --- | --- | --- | --- | --- |
| Leukocytes | 6.74 (0.03) | 6.63 (0.03) | 7.50 (0.06) | <0.001 | 6.61(0.03) | 7.03(0.04) | <0.001 | | 6.66(0.03) | 7.58(0.06) | <0.001 |
| Neutrophils | 3.91 (0.02) | 3.81 (0.02) | 4.57 (0.04) | <0.001 | 3.78(0.03) | 4.19(0.04) | <0.001 | | 3.84(0.02) | 4.64(0.05) | <0.001 |
| Lymphocytes | 2.04 (0.01) | 2.04 (0.01) | 2.08 (0.02) | 0.026 | 2.06(0.01) | 2.01(0.01) | 0.002 | | 2.04(0.01) | 2.07(0.02) | 0.141 |
| Monocytes | 0.54 (0.00) | 0.53 (0.00) | 0.57 (0.01) | <0.001 | 0.53(0.00) | 0.56(0.01) | <0.001 | | 0.53(0.00) | 0.58(0.01) | <0.001 |
| NLR | 2.06(0.01) | 2.00(0.01) | 2.41(0.03) | <0.001 | 1.96(0.01) | 2.28(0.02) | <0.001 | | 2.02(0.01) | 2.48(0.03) | <0.001 |
| PLR | 130.70(0.58) | 131.06(0.64) | 128.19(1.18) | 0.033 | 129.50(0.66) | 133.38(1.03) | 0.001 | | 130.92(0.61) | 128.32(1.56) | 0.118 |
| LMR | 4.09(0.02) | 4.11(0.02) | 3.93(0.04) | <0.001 | 4.20(0.02) | 3.86(0.04) | <0.001 | | 4.12(0.02) | 3.83(0.05) | <0.001 |

Abbreviations: T2DM, type 2 diabetes;

NLR, neutrophil to lymphocyte ratio (neutrophil/lymphocyte); PLR, platelet to lymphocyte ratio (platelet/lymphocyte); LMR, lymphocyte to monocyte ratio (lymphocyte/monocyte).

**Appendix Table 3** Interaction between baseline characteristics and RC in NHANES 2005-2018 (N=17,749).

| Variables | **T2DM** | |  | **Hypertension** | |  | **Hypertension&T2DM** | |  |
| --- | --- | --- | --- | --- | --- | --- | --- | --- | --- |
|  | OR (95%CI) | *P* value | P for interaction | OR (95%CI) | *P* value | P for interaction | OR (95%CI) | *P* value | P for interaction |
| **Sex** |  |  | **<0.001** |  |  | 0.568 |  |  | **0.007** |
| Male | 1.72(1.31,4.99) | <0.001 |  | 1.60(1.25,2.03) | <0.001 |  | 1.82(1.31,2.52) | <0.001 |  |
| Female | 3.46(2.40,4.99) | <0.001 |  | 1.79(1.36,2.34) | <0.001 |  | 3.34(2.28,4.88) | <0.001 |  |
| **Race** |  |  | 0.186 |  |  | **0.015** |  |  | 0.787 |
| Mexican American | 3.53(2.31,5.38) | <0.001 |  | 1.03(0.67,1.57) | 0.906 |  | 2.98(1.70,5.22) | <0.001 |  |
| Non-Hispanic Black | 3.26(2.08,5.13) | <0.001 |  | 1.21(0.87,1.69) | 0.243 |  | 3.00(1.75,5.16) | <0.001 |  |
| Non-Hispanic White | 1.98(1.44,2.73) | <0.001 |  | 1.87(1.50,2.34) | <0.001 |  | 2.14(1.53,2.98) | <0.001 |  |
| Other | 2.28(1.54,3.38) | <0.001 |  | 1.91(1.22,3.00) | 0.005 |  | 2.72(1.69,4.39) | <0.001 |  |
| **Education** |  |  | 0.320 |  |  | 0.121 |  |  | 0.628 |
| less than high school | 1.80(1.26,2.58) | 0.002 |  | 1.31(0.93,1.86) | 0.124 |  | 2.12(1.38,3.25) | <0.001 |  |
| high school | 2.65(1.75,4.01) | <0.001 |  | 1.61(1.11,2.34) | 0.013 |  | 2.83(1.83,4.37) | <0.001 |  |
| some college or above | 2.24(1.67,3.02) | <0.001 |  | 1.93(1.48,2.50) | <0.001 |  | 2.21(1.55,3.14) | <0.001 |  |
| **Marital status** |  |  | 0.243 |  |  | 0.968 |  |  | 0.656 |
| Married | 2.01(1.45,2.78) | <0.001 |  | 1.81(1.41,2.31) | <0.001 |  | 2.34(1.67,3.27) | <0.001 |  |
| Never married | 4.26(2.32,7.83) | <0.001 |  | 1.46(0.92,2.33) | 0.107 |  | 2.76(1.31,5.82) | 0.008 |  |
| Unmarried but have/had partner | 2.23(1.50,3.30) | <0.001 |  | 1.66(1.23,2.25) | 0.001 |  | 2.18(1.44,3.29) | <0.001 |  |
| **Smoking** |  |  | 0.271 |  |  | 0.451 |  |  | 0.223 |
| Former | 2.04(1.25,3.32) | 0.005 |  | 1.45(1.02,2.07) | 0.038 |  | 1.77(1.09,2.87) | 0.021 |  |
| Never | 2.73(2.05,3.64) | <0.001 |  | 1.75(1.32,2.32) | <0.001 |  | 2.90(2.08,4.05) | <0.001 |  |
| Now | 1.66(1.13,2.45) | 0.011 |  | 2.04(1.46,2.84) | <0.001 |  | 2.31(1.45,3.69) | <0.001 |  |
| **Current alcohol drinking** |  |  | 0.287 |  |  | 0.592 |  |  | 0.157 |
| No | 2.68(1.87,3.83) | <0.001 |  | 1.61(1.11,2.33) | 0.013 |  | 3.00(2.06,4.38) | <0.001 |  |
| Yes | 2.13(1.63,2.79) | <0.001 |  | 1.76(1.45,2.13) | <0.001 |  | 2.17(1.58,2.96) | <0.001 |  |
| **BMI levels** |  |  | 0.443 |  |  | **0.039** |  |  | **0.035** |
| Normal | 2.13(1.24,3.65) | 0.007 |  | 2.68(1.62,4.45) | <0.001 |  | 2.66(1.95,3.64) | 0.005 |  |
| Obese | 2.45(1.88,3.21) | <0.001 |  | 1.68(1.27,2.22) | <0.001 |  | 2.66(1.95,3.64) | <0.001 |  |
| Overweight | 1.95(1.36,2.80) | <0.001 |  | 1.46(1.10,1.94) | 0.010 |  | 1.55(1.05,2.28) | 0.027 |  |

*Adjustment for age, sex, race, education, marital status, PIR, smoking, alcohol, BMI levels, HEI, DII, and waist.

| **Appendix** **Table 4** Summary of simple mediation analyses for the relationships between mediator and outcomes, in NHANES 2005-2018 (n = 17749). | | | | |
| --- | --- | --- | --- | --- |
| Mediating Variable (M) | Direct effects (c′) | Indirect effect (a×b) | Total effects (c) | Proportion of mediation(%) |
| **Hypertension** | |  |  |  |
| White blood cell | 0.066*** | 0.008*** | 0.074*** | 10.79%*** |
| lymphocyte | 0.093*** | 0.001 | 0.093*** | 0.62% |
| Monocyte | 0.092** | 0.002*** | 0.094*** | 1.70%*** |
| Neutrophils | 0.088*** | 0.005*** | 0.093*** | 5.54%*** |
| NLR | 0.101*** | -0.001** | 0.100*** | -1.46%** |
| LMR | 0.125*** | -0.004* | 0.122*** | -3.20%* |
| PLR | 0.100*** | -0.000 | 0.100*** | -0.39% |
| **T2DM** | |  |  |  |
| White blood cell | 0.069*** | 0.012*** | 0.081*** | 14.46%*** |
| lymphocyte | 0.081*** | 0.001 | 0.084*** | 0.88% |
| Monocyte | 0.083*** | 0.0511*** | 0.0520*** | 1.75%** |
| Neutrophils | 0.078** | 0.007*** | 0.086*** | 8.65%*** |
| NLR | 0.087*** | -0.001*** | 0.086*** | -1.66%*** |
| LMR | 0.096*** | -0.002 | 0.095*** | -1.61% |
| PLR | 0.084*** | 0.001** | 0.085*** | 0.98%** |
| **Hypertension&T2DM** | |  |  |  |
| White blood cell | 0.054*** | 0.009*** | 0.062*** | 14.02%*** |
| lymphocyte | 0.058*** | 0.003** | 0.060*** | 4.36%** |
| Monocyte | 0.059*** | 0.001** | 0.060*** | 1.56%** |
| Neutrophils | 0.056*** | 0.006*** | 0.061*** | 9.38%*** |
| NLR | 0.063*** | -0.001*** | 0.062*** | -1.71%*** |
| LMR | 0.072*** | -0.002 | 0.069*** | -3.53% |
| PLR | 0.060*** | 0.001* | 0.061*** | 1.46%* |

*P* < 0.05; ** Indicate *P* < 0.01; *** Indicate *P* < 0.001.

**Abbreviations:** T2DM, type 2 diabetes;

NLR, neutrophil to lymphocyte ratio (neutrophil/lymphocyte); PLR, platelet to lymphocyte ratio (platelet/lymphocyte); LMR, lymphocyte to monocyte ratio (lymphocyte/monocyte).

**
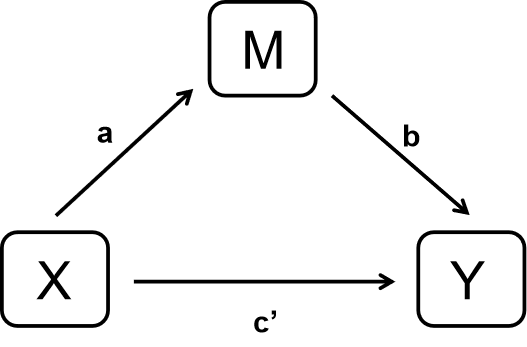
**

**Appendix Figure 1** Schematic of a simple mediation model.

Notes: coefficient a reflects the relationship between an independent variable (X) and a mediator variable (M), coefficient b reflects the relationship between M and a dependent variable (Y), and coefficient c’ reflects the direct effect of X on Y. The coefficients a and b were multiplied to compute the indirect effect (i.e., ab). The total effect is the sum of both the direct and mediated effects (i.e., c’ + ab). The proportion mediated was calculated by dividing the indirect effect (ab) by the total effect (c’ + ab)^1^.

1. Bellavia A, Zota AR, Valeri L, James-Todd T. Multiple mediators approach to study environmental chemicals as determinants of health disparities. *Environ Epidemiol.* 2018;2(2).
